# Complete response and HBsAg clearance of advanced hepatocellular carcinoma associated with hepatitis B by DEB−TACE combined systematic therapy: a case report

**DOI:** 10.3389/fimmu.2025.1676514

**Published:** 2025-11-20

**Authors:** Baiguo Xu, Yufeng Cui, Hao Cui, Ning Wang, Zhongsong Gao, Qing Ye

**Affiliations:** 1 Department of Gastroenterology and Hepatology, Tianjin Third Central Hospital, Tianjin, China; 2 Department of Gastroenterology and Hepatology, Tianjin University Central Hospital, Tianjin, China; 3 Department of Gastroenterology and Hepatology, Tianjin Institute of Hepatobiliary Disease, Tianjin, China; 4 Department of Gastroenterology and Hepatology, Tianjin Key Laboratory of Extracorporeal Life Support for Critical Diseases, Tianjin, China; 5 Department of Gastroenterology and Hepatology, Artificial Cell Engineering Technology Research Center, Tianjin, China

**Keywords:** hepatocellular carcinoma, local-regional therapy, DEB-TACE, immunotherapy, lenvatinib, tislelizumab, HBsAg clearance

## Abstract

**Background:**

Systemic therapy is the standard treatment for patients with hepatitis B-related hepatocellular carcinoma (HCC) and portal vein invasion. Emerging evidence suggests that the combination of localized regional therapy, multikinase inhibitors (MKIs), and immune checkpoint inhibitors (ICIs) yields promising outcomes for advanced HCC. ICIs not only effectively control HCC progression but may also facilitate a HBsAg clearance for hepatitis B-associated HCC patients, characterized by sustained HBV DNA (Hepatitis B Virus Deoxyribonucleic Acid)and HBsAg clearance. This report details a case of hepatitis B cirrhosis with multifocal intrahepatic HCC and portal vein involvement, where the patient achieved complete response (CR) and HBsAg clearance following treatment with lenvatinib (an MKI), tislelizumab (a PD-1 inhibitor), and localized regional therapy.

**Case summary:**

A 61-year-old male with hepatitis B-related cirrhosis and advanced HCC with portal vein involvement underwent drug-loaded microsphere hepatic arterial chemoembolization (DEB-TACE) treatment. Additionally, the patient received a combination therapy of lenvatinib and tislelizumab. Following three DEB-TACE sessions, six months of lenvatinib, and seven cycles of tislelizumab, the patient attained CR and achieved HBsAg clearance of hepatitis B, as evidenced by the sustained absence of HBV DNA and HBsAg.

**Conclusion:**

The concurrent administration of DEB-TACE, lenvatinib, and tislelizumab demonstrated efficacy in achieving a HBsAg clearance for hepatitis B in a patient presenting with advanced HCC and portal vein tumor thrombosis. These findings suggest that a localized regimen incorporating MKIs and ICIs holds promise as a therapeutic approach for select cases of HBV-HCC, potentially leading to a HBsAg clearance of hepatitis B. Large-scale studies are warranted to validate these findings.

## Introduction

Primary liver cancer is often asymptomatic in its early stages, resulting in delayed diagnosis (1). Hepatocellular carcinoma (HCC) with portal vein tumor thrombosis (PVTT) represents an aggressive phenotype associated with poor prognosis. PVTT is present in approximately 30–44% of advanced HCC cases, indicating advanced disease with limited treatment options (2). Patients with HCC and macroscopic PVTT experience a median survival time of 6–9 months without intervention, with a one-year overall survival rate ranging from 20% to 47% (3–5). Systemic therapy is the standard treatment for HCC with PVTT classified as Barcelona Clinical Liver Cancer (BCLC) stage C (6, 7). Recent studies indicate that the combination of local-regional therapy with systemic therapy enhances objective response rate and overall survival. In recent years, artificial intelligence (AI) and image segmentation technologies have provided new tools for HCC diagnosis and treatment, optimizing lesion identification, efficacy evaluation, and treatment planning (8). For example, image segmentation can accurately quantify tumor volume and the extent of portal vein involvement, providing objective evidence for selecting local-systemic combination therapy regimens in patients with BCLC stage C of HCC. However, the optimal combination of systemic treatments for HCC with PVTT remains contentious, as treatment approaches are often guided by individual clinical experience. Immune checkpoint inhibitors (ICIs) have demonstrated potential in halting HCC progression, recurrence, and metastasis by enhancing the body’s anti-tumor immune response (9). Nevertheless, uncertainties persist regarding the efficacy of ICIs against hepatitis B virus (HBV) in HBV-infected individuals (10). While ICIs may enhance the immune response to HBV, they could also contribute to elevated HBV viral load, underscoring the need to further explore of the potential of ICIs in HBV-infected individuals (11).

## Case report

**Figure 1:**
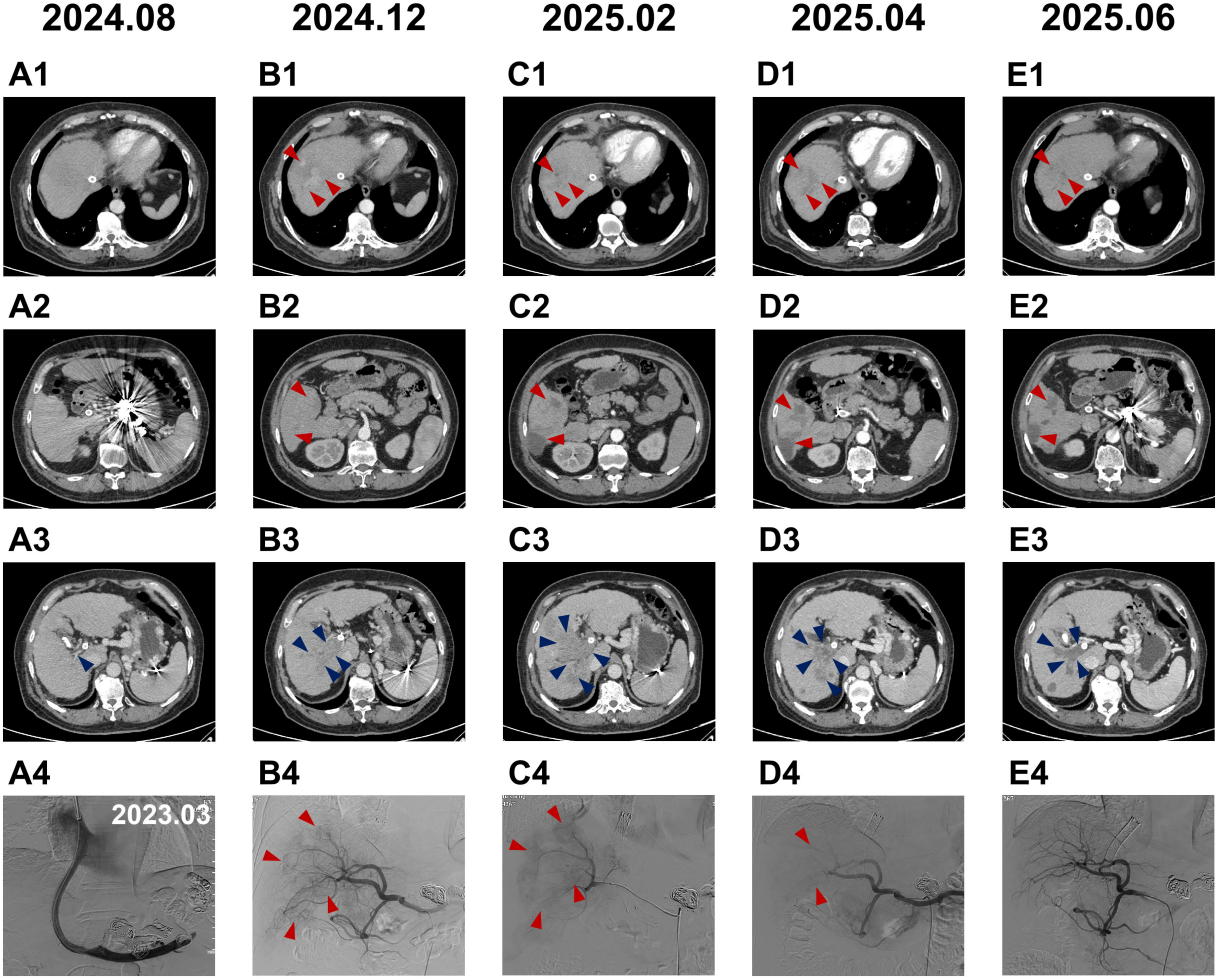
Representative abdominal contrast-enhanced CT and DSA images during clinical treatment. **(A1, A2)** August 2024, no intrahepatic malignant lesions identified;**(A3)** Portal vein appeared slender with no intraluminal tumor thrombus; **(A4)** TIPSS combined with gastric variceal embolization was performed in March 2023. **(B1, B2)** December 2024, first detection of multiple hepatocellular carcinomas in the right hepatic lobe (indicated by red arrows); **(B3)** Tumor thrombus formation in the right portal vein and its tributaries (indicated by blue arrows); **(B4)** First DEB-TACE procedure, DSA angiography showed contrast staining of multiple hepatocellular carcinomas in the right hepatic lobe along with a tumor thrombus in the right portal vein (indicated by red arrows). Intraoperative embolization was performed using 1 g of drug-eluting microspheres. **(C1, C2)** February 2025, post-treatment changes in right hepatic lobe hepatocellular carcinoma with hypodense necrotic areas (indicated by red arrows); **(C3)** Progressive enlargement of tumor thrombus in the right portal vein and its tributaries compared with prior images (indicated by blue arrows); **(C4)** Second DEB-TACE procedure, DSA angiography showed contrast staining of right hepatic lobe hepatocellular carcinoma and right portal vein tumor thrombus (indicated by red arrows). Intraoperative embolization was performed with 1 g of drug-eluting microspheres. **(D1, D2)** April 2025, post-treatment changes of right hepatic lobe hepatocellular carcinoma demonstrating further increased necrotic areas (indicated by red arrows); **(D)** Post-treatment changes in tumor thrombus in the right portal vein and its tributaries revealing clear necrotic regions (indicated by blue arrows); **(D4)** Third DEB-TACE procedure, DSA angiography showed faint contrast staining of residual hepatocellular carcinoma in the right hepatic lobe and right portal vein tumor thrombus (indicated by red arrows). An appropriate amount of drug-eluting microspheres was injected for embolization. **(E1, E2)** June 2025, post-treatment changes of right hepatic lobe hepatocellular carcinoma with complete tumor necrosis, evaluated as CR according to mRECIST criteria (indicated by red arrows); **(E3)** Post-treatment changes in tumor thrombus within the right portal vein and its tributaries with complete necrosis (indicated by blue arrows); **(E4)** DSA angiography showed no contrast staining of intrahepatic hepatocellular carcinoma or tumor thrombus. TIPSS, Transjugular Intrahepatic Portosystemic Shunt; DEB-TACE, drug-eluting bead transarterial chemoembolization; mRECIST, modified Response Evaluation Criteria in Solid Tumors; DSA, digital subtraction angiography;CR, complete response.

The patient is a 61-year-old male worker with no family history of hereditary liver diseases or malignancies, a 10-plus-year smoking history (now quit; previously smoked 4–5 cigarettes daily), and no alcohol use history, who has a medical history of hepatitis B-related cirrhosis, cirrhosis-associated esophagogastric variceal bleeding, and prior transjugular intrahepatic portosystemic shunt (TIPSS) treatment—specifically, he underwent TIPSS in March 2023 for cirrhosis-related esophagogastric variceal bleeding, and postoperatively, he began and maintained antiviral therapy with Tenofovir Dipivoxil Fumarate Tablets (300 mg once daily), with his HBV DNA levels stably maintained at < 20 IU/mL—and was hospitalized in December 2024 due to recurrent abdominal distension lasting more than one week; his previous abdominal enhanced computed tomography (CT) findings are displayed in **Figures 1A1-A4**.

On admission, vital signs were stable (blood pressure 135/85 mmHg, heart rate 78 bpm, respiratory rate 18 breaths/min, temperature 36.8°C). Abdominal examination: soft and flat, no tenderness/rebound tenderness, liver/spleen not palpable below costal margin, shifting dullness negative, bowel sounds normal (4–5 times/min). No lower extremity edema. Neurological examination: alert, no asterixis (consistent with absence of hepatic encephalopathy at admission).

An enhanced CT scan of the chest and abdomen revealed multiple enhancing nodules in the right lobe of the liver, indicative of liver cancer (LR-5), with the largest lesion measuring 4.2 × 3.8 cm. Additionally, there was evidence of tumor thrombus formation in the right portal vein and its branches (LR-TIV), with stent involvement, consistent with a diagnosis of HCC and PVTT (**Figures 1B1-B3**). The patient was classified as BCLC stage C.

Laboratory analyses yielded the following results: white blood cell count of 2.80 × 10^9^/L (normal range: 3.50 - 9.50 × 10^9^/L), red blood cell count of 4.14 × 10^12^/L (normal range: 3.80 - 5.10 × 10^12^/L), platelet count of 146 × 10^9^/L (normal range: 125 - 350 × 10^9^/L), and an International Normalized Ratio of 1.34 (0.85 - 1.50). Liver function tests indicated serum albumin levels of 38.4 g/L (normal range: 35 - 50g/L), alanine aminotransferase at 53 U/L(normal range: 9–50 U/L), aspartate aminotransferase at 44 U/L(normal range: 13–35 U/L), alkaline phosphatase at 161 U/L(normal range: 50–135 U/L), gamma-glutamyl transferase at 220 U/L(normal range: 7–45 U/L), and total bilirubin at 27.8 µmol/L(normal range: 3.4–20.5 μmol/L). Additionally, alpha-fetoprotein levels were recorded at 3.04 ng/mL (normal range: < 7 ng/mL). HBV markers included HBsAg at 58.92 IU/mL, HBeAg at 0.29, HBeAb at 0.03, HBcAb at 6.25, and HBV DNA levels at 23 IU/mL. Tenofovir Dipivoxil Fumarate Tablets (300 mg QD) were continued for antiviral therapy.

Diagnostic reasoning and differential diagnosis: Integrated HBV history (HBsAg positive), Contrast-enhanced CT features (arterial-phase enhancement/venous-phase washout and PVTT) to confirm HBV-HCC. Differentiation from liver metastases: The patient had no history of other malignancies; AFP was within the normal range but HBsAg was positive; CT showed lesions localized to the right hepatic lobe with PVTT—findings consistent with HBV-related HCC; Differentiation from hepatic hemangioma: Hemangiomas exhibit “early peripheral enhancement and delayed centripetal filling” on dynamic contrast imaging, whereas the lesions in this case showed arterial-phase enhancement, venous-phase washout, and associated PVTT, ruling out hemangioma.

The patient was BCLC stage C (standard systemic therapy), but with Child-Pugh A liver function and strong treatment willingness, we opted for local + systemic therapy. DEB-TACE was selected for PVTT and right hepatic lesions (precise embolization for thorough necrosis); lenvatinib + tislelizumab were chosen for domestic accessibility and the patient’s financial affordability. Adjustments (2nd/3rd DEB-TACE after PD) were based on efficacy.

The patient underwent drug-eluting bead transarterial chemoembolization (DEB-TACE) using 1 g of microspheres (CalliSpheres, Hengrui Medicine, China; diameter range: 300–500 μm) loaded with 40 mg of epirubicin (**Figure 1B4**). No platinum-based compounds were administered. Concurrently, the patient received combination therapy with lenvatinib (12 mg once daily) and tislelizumab (200 mg every three weeks).

Eight weeks following the initial interventional therapy, abdominal contrast-enhanced CT demonstrated post-treatment changes in the right hepatic lobe HCC, with hypodense necrotic areas identified within the lesion (**Figures 1C1, C2**). However, progressive enlargement of the tumor thrombus was noted in the right portal vein and its tributaries compared to prior imaging (**Figure 1C3**). According to the modified Response Evaluation Criteria in Solid Tumors (mRECIST), the disease was assessed as progressive disease. A second DEB-TACE procedure was subsequently performed using 1 g of drug-eluting microspheres (diameter: 100–300 μm) loaded with 40 mg of epirubicin for embolization (**Figure 1C4**).

At 16 weeks after the first DEB-TACE, a follow-up upper abdominal contrast-enhanced CT revealed further progression of post-treatment changes in the right hepatic lobe HCC, with a marked increase in necrotic areas but residual lesions(**Figures 1D1-D2**). Subsequent post-treatment alterations were also observed in the PVTT involving the right portal vein and its tributaries, with evident necrotic regions detected (**Figure 1D3**). The response was evaluated as Partial Response (PR) based on the mRECIST criteria, prompting a third DEB-TACE. Digital subtraction angiography (DSA) demonstrated faint contrast staining of residual HCC in the right hepatic lobe and the right portal vein tumor thrombus. During this procedure, 0.4 g of drug-eluting microspheres (diameter: 100–300 μm, loaded with 40 mg of epirubicin per 1 g microspheres) were administered for embolization (**Figure 1D4**).

Twenty-four weeks post-initial DEB-TACE, upper abdominal contrast-enhanced CT displayed post-treatment changes in the right hepatic lobe HCC with complete tumor necrosis (**Figures 1E1, E2**). Post-treatment changes were observed in the PVTT within the right portal vein and its tributaries, confirming complete necrosis of the tumor thrombus (**Figure 1E3**). No new tumor lesions were identified, and the response was assessed as complete response (CR) based on mRECIST criteria. DSA imaging indicated no contrast staining of intrahepatic HCC or tumor thrombus (**Figure 1E4**), demonstrating significant improvement in hepatic blood flow compared with prior assessments, thus negating the need for further embolization or chemotherapy.

**Figure 2:**
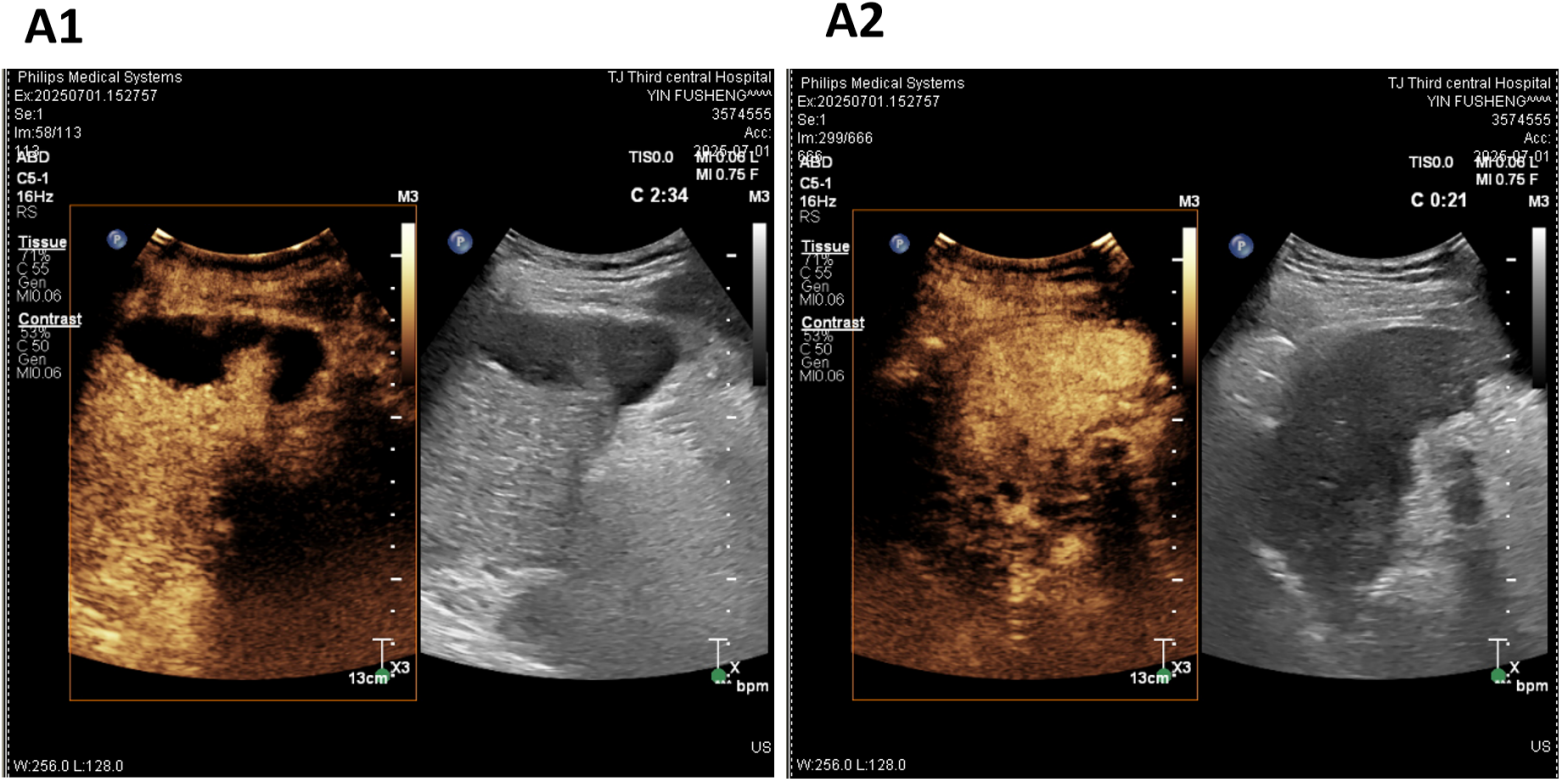
Contrast-enhanced ultrasound (CEUS) images after radiological complete response. **(A1, A2)** CEUS (July 1, 2025): 1.5 ml of SonoVue was administered via bolus injection through the left median cubital vein. Starting from 12 seconds into the hepatic arterial phase, no contrast agent enhancement was observed in multiple intrahepatic lesions during the arterial phase, portal venous phase, or delayed phase, with clear boundaries. The embolus in the right portal vein showed no enhancement in the arterial phase, contrast agent inflow during the portal venous phase, and hypoenhancement in the delayed phase. No additional washout lesions were detected throughout the liver during the delayed phase scanning. CEUS, Contrast-Enhanced Ultrasound.

As of July 1, 2025 (25 weeks after the first DEB-TACE), follow-up contrast-enhanced abdominal ultrasound confirmed the absence of viability in both the HCC lesion and PVTT, with no new tumor lesions detected (**Figures 2A1, A2**). Regular imaging surveillance is planned every 8–12 weeks thereafter.

The patient exhibited consistent adherence to the treatment regimen, reflecting strong therapeutic motivation, expressing satisfaction with the treatment outcome. The patient received TDF (300 mg once daily) and continued uninterrupted through the latest follow-up (July 2025, 25 weeks post-first DEB-TACE). We confirmed that antiviral therapy was not discontinued post-systemic/locoregional treatment, as sustained antiviral suppression is critical for preventing HBV reactivation during immunotherapy and maintaining HBsAg clearance.

**Figure 3:**
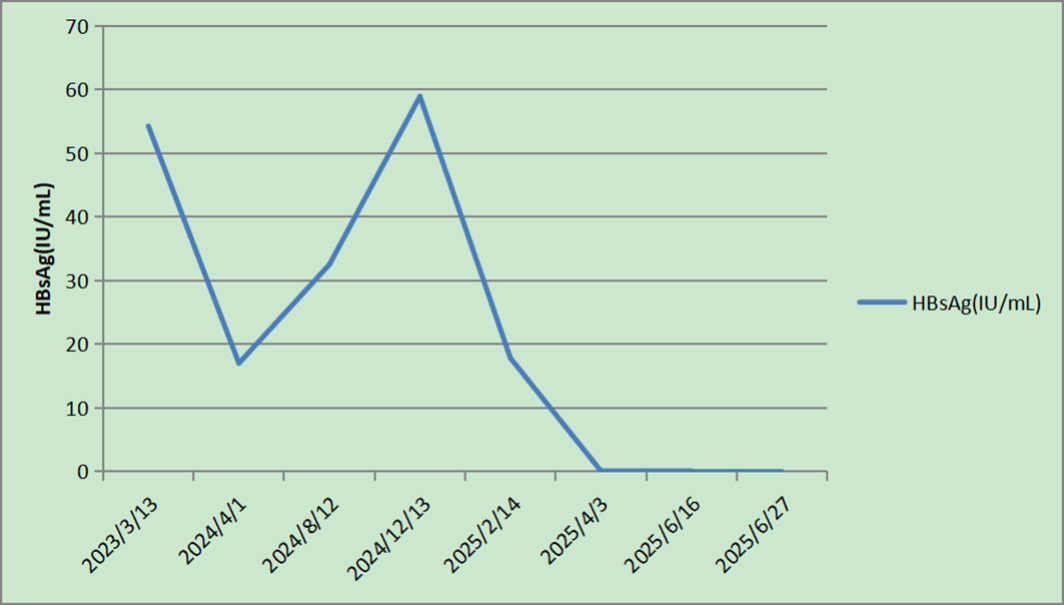
Changes in HBsAg levels during treatment. Figure shows a sustained decrease in HBsAg levels below the detection limit after the initiation of combination therapy.

Continuous administration of lenvatinib and tislelizumab has been maintained, along with scheduled chest and abdominal enhanced CT scans performed every 8–12 weeks. As of July 2, 2025, the patient had sustained CR without any tumor recurrence. Throughout this observation period, alpha-fetoprotein levels remained within the normal range, while HBV DNA levels decreased to < 20 IU/mL, and HBsAg became negative, although serological conversion (HBsAb positive) had not been achieved. Trends in HBsAg levels are illustrated in **Figure 3**. In December 2024, the patient received the first DEB-TACE plus combination therapy with lenvatinib and tislelizumab → in February 2025, disease progression (PD) was noted, and a second DEB-TACE was performed → in April 2025, PR was achieved, and a third DEB-TACE was administered → in June 2025, CR was confirmed, and a summary of the treatment schedule is provided in **Table 1**.

**Table 1:** Treatment timeline of the patient.

Date	Locoregional therapy or ICIs	MKIs	(mRECIST)
2024.12.17	DEB-TACE	L	
2025.1.14	T		
2025.2.6	T		
2025.2.18	DEB-TACE		PD
2025.2.28	T		
2025.3.21	T		
2025.4.10	DEB-TACE		PR
2025.4.27	T		
2025.5.20	T		
2025.6.10	T		
2025.6.16	DSA		CR

MKIs, multi-kinase inhibitors; ICIs, immune checkpoint inhibitors; mRECIST, the modified Response Evaluation Criteria in Solid Tumors; DEB-TACE, drug-eluting bead transarterial chemoembolization; L, Lenvatinib; T, Tislelizumab; PD, Progressive Disease;PR, partial response; CR, complete response.

The patient exhibited good compliance with the prescribed interventions, underwent regular follow-up assessments, and adverse events were graded per the Common Terminology Criteria for Adverse Events (CTCAE) Version 5.0. Grade 2 hypertension (managed with oral amlodipine besylate tablets, dosage tailored to baseline blood pressure and tolerance) and grade 1 diarrhea (addressed with supportive measures like oral rehydration salts and loperamide) were well tolerated without treatment interruption, which the patient confirmed: “Mild diarrhea and hypertension during treatment were well-controlled with medication and did not affect my daily life.” After achieving CR, the patient resumed light office work, feels optimistic about the prognosis, and will continue regular follow-up and medication adherence to maintain outcomes.

**Figure 4:**
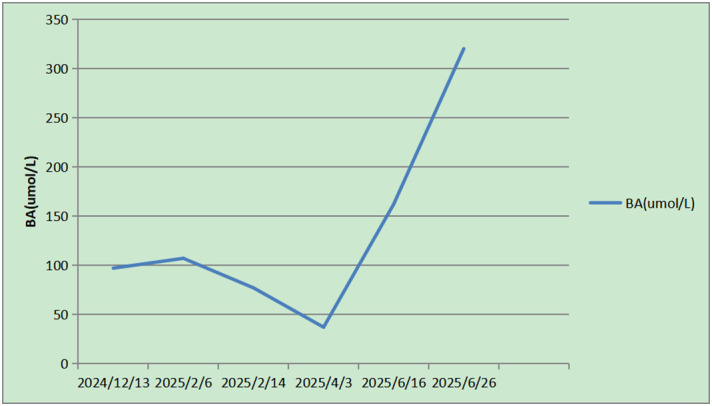
Changes in blood ammonia (BA) levels during treatment. Figure shows a continuous decrease in BA levels after the occurrence of HCC, followed by a significant increase. This trend was consistent with the progression of PVTT involvement and exhibited a corresponding pattern of response from partial response to complete response after treatment. BA, blood ammonia; PVTT, portal vein tumor thrombus.

Interestingly, after the resolution of the PVTT, the patient experienced repeated episodes of grade 1–3 hepatic encephalopathy (3–5 episodes) without an apparent precipitant. The curve of blood ammonia change is shown in **Figure 4**. Following the implementation of enhanced dietary management, intestinal acidification, promotion of stool elimination, and supplementation with branched-chain amino acids and ornithine aspartate, the symptoms improved, and the patient’s condition stabilized.

## Discussion

HCC often has an insidious onset, rendering most patients unsuitable for surgical resection at the time of diagnosis (12, 13). In mainland China, 55% of HCC cases are diagnosed at BCLC stage C, which is associated with a poor prognosis (14). In current clinical practice, systemic therapy is considered the main treatment approach for these patients (15). Approved systemic therapies include TKIs and ICIs. Studies have shown synergistic antitumor effects between TKIs and ICIs (16), although local-regional treatments such as TACE are generally not recommended for patients with BCLC stage C of HCC. TACE, including DEB-TACE, remains the primary treatment option for patients diagnosed at BCLC stage B (17). Shi et al. have demonstrated that DEB-TACE exhibits superior efficacy and survival outcomes compared to conventional TACE in patients with BCLC stages A and B (18). Meta-analyses also support DEB-TACE, indicating improved tumor response rates (19, 20). However, the evidence supporting its benefit in BCLC stage C HCC is limited, and multicenter randomized controlled trials are currently lacking.

TACE induces hepatic hypoxia and triggers the expression of vascular endothelial growth factor (VEGF), which, in combination with TKIs and ICIs, may exert a synergistic therapeutic effect. This case underscores the critical importance of integrating local-regional therapy, TKIs, and ICIs for the management of HCC characterized by intrahepatic multiple lesions and PVTT. The patient in this report received DEB-TACE in combination with lenvatinib and tislelizumab—a strategy not typically recommended by current clinical guidelines—yet it resulted in favorable outcomes. To our knowledge, this is the first documented case of advanced HCC that includes intrahepatic multiple lesions and PVTT, demonstrating CR, HBsAg clearance, and improved TIPSS stent blood flow following treatment with DEB-TACE in combination with lenvatinib and tislelizumab.

This modification in treatment strategy illustrates the adaptability required in clinical practice and provides a valuable reference for future management of similar cases- a subgroup that is often excluded from standard clinical trials and lacks clear management recommendations in current guidelines (e.g., the 2022 Chinese Guidelines for Chronic Hepatitis B (21); AASLD 2023 HCC Guidance (6). Furthermore, this case highlights the necessity for vigilance regarding the potential increase in blood ammonia levels and the occurrence of hepatic encephalopathy, which may arise from the restoration of portal vein blood flow after necrosis of the PVTT. It is recommended to strengthen the monitoring of such patients and give preventive medication for hepatic encephalopathy.

Since the pivotal REFLECT study in 2018, lenvatinib has been established as a first-line treatment for HCC. Mechanistically, lenvatinib has been found to inhibit monocyte and macrophage activity within tumors, enhance T lymphocyte activation, and modulate immune responses (22). Additional evidence suggests that TKIs such as lenvatinib may improve tumor immunosuppressive pathways, thereby enhancing the efficacy of ICIs (23).

Tislelizumab is a structurally optimized, humanized anti-PD-1 monoclonal antibody developed independently in China. It can reverse T cell immunosuppression and enhance antitumor activity through high-affinity binding to the PD-1 receptor, thus blocking its interaction with PD-L1 and PD-L2. The Fc segment of tislelizumab has been specifically engineered (IgG4 variant) to eliminate the ability to bind FcγR, thereby averting T-cell depletion caused by antibody-dependent cellular phagocytosis (24). The global, multicenter phase 3 clinical study (RATIONALE 301), which involved approximately 80% of patients at BCLC stage C, revealed a median overall survival of 15.9 months for tislelizumab. This treatment demonstrated a 15% reduction in the risk of mortality and a 1.8-month increase in median overall survival compared to sorafenib, alongside a trend towards survival benefit. Notably, the overall response rate for tislelizumab was 14.3%, with nearly 3% of patients (11) achieving a CR and a median duration of response of 36.1 months, highlighting its significant efficacy in advanced HCC (25).

In the present case, the patient achieved CR after three DEB-TACE sessions, approximately six months of lenvatinib treatment, and seven cycles of tislelizumab. These findings reinforce the efficacy of combining DEB-TACE with lenvatinib and tislelizumab for the treatment of HCC.

Notably, while the dual effects of tislelizumab — contributing to both antitumor activity and antiviral control in HBV-HCC — are primarily mediated through three key pathways, these mechanisms directly align with the favorable virological outcomes observed in our patient, who achieved a complete virological response (HBV DNA < 20 IU/mL) and HBsAg clearance [a widely recognized ideal endpoint for HBV infection (26)]. This phenomenon is not accidental. It has been clearly proposed in existing studies that HCC patients, due to their high prevalence of chronic HBV infection, long-term need for ICI treatment, and enrichment of HBV-specific exhausted T cells in the liver microenvironment, are an ideal study population for evaluating the anti-HBV efficacy of ICIs (27), which provides population-based evidence for the “dual tumor-viral response” observed in this case.

The dual effects of tislelizumab are primarily mediated through the following pathways: First, in reversing HBV-specific T-cell exhaustion: within the HBV-HCC tumor microenvironment, HBV-specific CD4+ T cells highly express exhaustion markers (e.g., PD-1, Tim-3), impairing their IFN-γ secretion; tislelizumab binds PD-1 with high affinity (its Fc segment is engineered to avoid T-cell phagocytosis (25) to block the PD-1/PD-L1 pathway, restoring the cytotoxic activity of HBV-infected hepatocytes (28). Mechanistically, this T-cell function restoration is consistent with the core mechanism of PD-1/PD-L1 inhibitors in chronic HBV infection: blocking the PD-1/PD-L1 axis can reverse the exhausted state of HBV-specific CD8+ T cells, restore their cytokine secretion (e.g., IFN-γ, TNF-α) and cytotoxic capacity, and thereby enhance the clearance efficiency of HBV-infected hepatocytes (29), which further supports the link between PD-1 inhibition and HBsAg clearance in our case.

Second, in modulating the tumor microenvironment’s immune balance: tislelizumab promotes the proliferation of tumor-infiltrating CD8+ T cells while inhibiting regulatory T cells (Treg) and M2-type macrophages, reducing immunosuppressive factors (e.g., IL-10, TGF-β) to enhance antitumor immunity and indirectly suppress HBV replication (30). Third, in exerting a synergistic effect with multikinase inhibitors: lenvatinib blocks the VEGF pathway to inhibit tumor angiogenesis and reduce myeloid-derived suppressor cell (MDSC) infiltration, enhancing tislelizumab’s immune activation to form an “anti-angiogenesis-immune activation” synergy (23). The immunosuppressive tumor microenvironment in HBV-HCC is particularly amenable to PD-1 inhibitor-mediated reversal, as supported by evidence that ICIs reduce HBV DNA levels in HBV-HCC patients without HBV reactivation (31), and basic studies specific to tislelizumab (the ICI used in our case) demonstrate its ability to induce high PD-L1 expression in HBV-infected tissue microenvironments and alleviate immunosuppression more efficiently via its high affinity for PD-1 (32). This aligns with Hoogeveen et al.’s finding that PD-1 inhibitors revert the “depleted phenotype” of HBV-specific T cells (e.g., reduced PD-1, Tim-3 expression), restoring their capacity to secrete IFN-γ and lyse infected hepatocytes (28). However, clinical data linking ICIs to HBsAg clearance remain limited to individual cases (including ours), underscoring the need for further clinical trials to clarify ICIs’ specific impact on HBV load and their potential to consistently drive HBsAg clearance in HBV-HCC populations.

Our patient’s baseline HBsAg level (58.92 IU/mL, < 100 IU/mL) aligns with a multi-cohort real-world study, which included 162 cancer patients receiving ICI treatment and confirmed that baseline HBsAg < 100 IU/mL was an independent predictor of HBsAg clearance (HR = 6.274, p = 0.028); notably, the 24-month cumulative HBsAg clearance rate in this subgroup reached 38.4%, significantly higher than that in patients with baseline HBsAg ≥ 100 IU/mL (33).

Although ICIs have shown promise in the treatment of HBV-HCC, concerns persist regarding potential side effects, particularly the risk of HBV reactivation (34). The mechanism underlying HBV reactivation is primarily associated with immune system modulation induced by ICIs, which may transition the virus from a latent to an active state, potentially resulting in severe liver function damage (35).Notably, reactivation risk is significantly reduced in patients receiving continuous antiviral prophylaxis: Shao et al. showed that prophylactic use of nucleos(t)ide analogs (NAs) such as tenofovir dipivoxil fumarate (TDF) or entecavir (ETV) lowers reactivation risk to <1% (31), which is consistent with the 2022 Chinese Guidelines for Chronic Hepatitis B recommendation that “all HBV-HCC patients receiving ICIs should receive lifelong NA prophylaxis (21).

Interestingly, after the resolution of the PVTT, the patient experienced repeated episodes of grade 1–3 hepatic encephalopathy. The core mechanism underlying HE after PVTT necrosis is ammonia metabolism disorders associated with restored portal blood flow: Changes in portal hemodynamics: After PVTT embolization, blood flow in the main portal vein and its branches is obstructed, increasing the flow of portosystemic shunts (e.g., TIPSS stents). Following PVTT necrosis, portal blood flow is suddenly restored; however, ammonia derived from the intestine (which enters the liver via the portal vein) cannot be fully detoxified due to impaired urea synthesis in cirrhotic hepatocytes, leading to elevated blood ammonia; Intestinal microbiota dysbiosis: DEB-TACE and targeted therapy may damage the intestinal mucosal barrier, causing intestinal microbiota dysbiosis (e.g., increased ammonia-producing bacteria), which further enhances ammonia production; Increased blood-brain barrier (BBB) permeability: In cirrhosis, BBB integrity is compromised, allowing elevated blood ammonia to readily enter the brain parenchyma. This inhibits the activity of glutamatergic neurons in the central nervous system, triggering HE (36).

Despite these promising results, uncertainty remains about the optimal strategy for selecting TACE regimens and combining TKIs with ICIs. This case highlights the success of an unconventional approach involving DEB-TACE, tislelizumab, and lenvatinib in the management of HCC with PVTT. These findings provide new insights and potential avenues for treating advanced HCC, yet it has inherent limitations as a single case report. First, the favorable outcomes (CR and HBV HBsAg clearance) were observed in only one patient with stage C of BCLC, so individual variability may exist and the results cannot be generalized to all similar patients; validation in large multicenter cohorts is needed to confirm the regimen’s reproducibility. Second, the lack of pretreatment biomarker analysis (e.g., PD-L1 expression, CD8+ T-cell infiltration) limits mechanistic interpretation of the therapeutic response. Though biomarker-driven methods (e.g., PD-L1 staining) show potential for predicting immune checkpoint inhibitor (ICI) responses in HCC (37), and we have discussed the controversial predictive value of PD-L1 [noting its insufficiency for reliable ICI efficacy prediction in HCC (38)], the absence of baseline data still hinders understanding of the immune microenvironment-treatment efficacy interplay. Third, efficacy assessment relied on traditional radiological interpretation (e.g., contrast-enhanced CT, DSA) rather than quantitative image segmentation, which could improve evaluation precision in future studies. Fourth, follow-up (25 weeks post-initial DEB-TACE as of July 2025) is insufficient; longer surveillance is required to monitor tumor recurrence, HBV reactivation, and late adverse events, ensuring the durability of CR and functional cure.

## Conclusions

In this case, a patient with hepatitis B-related cirrhosis complicated by multiple intrahepatic HCC lesions with PVTT was effectively managed and achieved a HBsAg clearance for hepatitis B through a combination of local-regional therapy, multi-target TKIs, and ICIs. Given the current lack of consensus and limited evaluation surrounding the use of DEB-TACE in combination with lenvatinib and tislelizumab for patients with HBV-associated HCC and PVTT, our findings suggest that this combination represents a viable treatment option. Nevertheless, the attainment of HBsAg clearance for HBV-associated HCC via ICIs varies significantly among different patient cohorts, necessitating further exploration of individual patient differences in the future. This includes an examination of immune microenvironment characteristics, tumor heterogeneity, and hepatitis B virus load, to better identify HBV-associated HCC patients who stand to benefit from this therapeutic approach.

## Data Availability

The raw data supporting the conclusions of this article will be made available by the authors, without undue reservation.

## References

[1] Gu W Yang Y Liu J Xue J Zhao H Mao L . Tumor-derived exosomes promote macrophages M2 polarization through miR-1-3p and regulate the progression of liver cancer. Mol Immunol. (2023) 162:64–73. doi: 10.1016/j.molimm.2023.08.006, PMID: 37657187

[2] Kokudo T Hasegawa K Matsuyama Y Takayama T Izumi N Kadoya M . Survival benefit of liver resection for hepatocellular carcinoma associated with portal vein invasion. J Hepatol. (2016) 65:938–43. doi: 10.1016/j.jhep.2016.05.044, PMID: 27266618

[3] Zhou J Tang ZY Wu ZQ Zhou XD Ma ZC Tan CJ . Factors influencing survival in hepatocellular carcinoma patients with macroscopic portal vein tumor thrombosis after surgery, with special reference to time dependency: a single-center experience of 381 cases. Hepatogastroenterology. (2006) 53:275–80., PMID: 16608039

[4] Ikeda M Okusaka T Ueno H Morizane C Iwasa S Hagihara A . Hepatic arterial infusion chemotherapy with epirubicin in patients with advanced hepatocellular carcinoma and portal vein tumor thrombosis. Oncology. (2007) 72:188–93. doi: 10.1159/000112805, PMID: 18097170

[5] Sui WF Li JY Fu JH . Transarterial chemoembolization plus stent placement for hepatocellular carcinoma with main portal vein tumor thrombosis: A meta-analysis. World J Clin Oncol. (2024) 15:447–55. doi: 10.5306/wjco.v15.i3.447, PMID: 38576592 PMC10989260

[6] Singal AG Llovet JM Yarchoan M Mehta N Heimbach JK Dawson LA . AASLD Practice Guidance on prevention, diagnosis, and treatment of hepatocellular carcinoma [published correction appears in Hepatology. 2023 Dec 1;78(6):E105. doi: 10.1097/HEP.0000000000000621. Hepatology. (2023) 78:1922–65. doi: 10.1097/HEP.0000000000000466, PMID: 37199193 PMC10663390

[7] Reig M Forner A Rimola J Ferrer-Fàbrega J Burrel M Garcia-Criado Á . BCLC strategy for prognosis prediction and treatment recommendation: The 2022 update. J Hepatol. (2022) 76:681–93. doi: 10.1016/j.jhep.2021.11.018, PMID: 34801630 PMC8866082

[8] Mohapatra RK Jolly L Dakua SP . Advancing explainable AI in healthcare: Necessity, progress, and future directions. Comput Biol Chem. (2025) 119:108599. doi: 10.1016/j.compbiolchem.2025.108599, PMID: 40743677

[9] Ghavimi S Apfel T Azimi H Persaud A Pyrsopoulos NT . Management and treatment of hepatocellular carcinoma with immunotherapy: a review of current and future options. J Clin Transl Hepatol. (2020) 8:168–76. doi: 10.14218/JCTH.2020.00001, PMID: 32832397 PMC7438354

[10] Han JW Park SH . Advances in immune checkpoint inhibitors for hepatocellular carcinoma. J Liver Cancer. (2021) 21:139–45. doi: 10.17998/jlc.2021.09.24, PMID: 37383085 PMC10035682

[11] Huang J Cai M He X . Serum potassium levels and prognosis in HBV-associated decompensated cirrhosis. J Clin Lab Anal. (2021) 35:e23775. doi: 10.1002/jcla.23775, PMID: 33951234 PMC8183925

[12] Zhou M Wang H Zeng X Yin P Zhu J Chen W . Mortality, morbidity, and risk factors in China and its provinces, 1990–2017: a systematic analysis for the global burden of disease study 2017. Lancet. (2017) 394:1145–58. doi: 10.1016/s0140-6736(19)30427-1, PMID: 31248666 PMC6891889

[13] Bray F Ferlay J Soerjomataram I Siegel RL Torre LA Jemal A . Global cancer statistics 2018: GLOBOCAN estimates of incidence and mortality worldwide for 36 cancers in 185 countries. CA Cancer J Clin. (2018) 68:394–424. doi: 10.3322/caac.21492, PMID: 30207593

[14] Park J Chen M Colombo M Roberts LR Schwartz M Chen P . Global patterns of hepatocellular carcinoma management from diagnosis to death: the BRIDGE study. Liver Int. (2015) 35:2155–66. doi: 10.1111/liv.12818, PMID: 25752327 PMC4691343

[15] Raoul JL Schirmacher P Vilgrain V . Corrigendum to EASL clinical practice guidelines: management of hepatocellular carcinoma. J Hepatol. (2018) 69:182–236. doi: 10.1016/j.jhep.2019.01.020, PMID: 29628281

[16] Roland CL Dineen SP Lynn KD Sullivan LA Dellinger MT Sadegh L . Inhibition of vascular endothelial growth factor reduces angiogenesis and modulates immune cell infiltration of orthotopic breast cancer xenografts. Mol Cancer Ther. (2009) 8:1761–71. doi: 10.1158/1535-7163.mct-09-0280, PMID: 19567820

[17] Llovet JM Real MI Montaña X Planas R Coll S Aponte J . Arterial embolisation or chemoembolisation versus symptomatic treatment in patients with unresectable hepatocellular carcinoma: a randomised controlled trial. Lancet. (2002) 359:1734–9. doi: 10.1016/s0140-6736(02)08649-x, PMID: 12049862

[18] Shi Z Wang D Kang T Yi R Cui L Jiang H . Comparison of CalliSpheres(^®^) microspheres drug-eluting beads and conventional transarterial chemoembolization in hepatocellular carcinoma patients: a randomized controlled trial. Radiol Oncol. (2023) 57:70–9. doi: 10.2478/raon-2023-0001, PMID: 36794998 PMC10039469

[19] Xie Z Wang X Peng Y Zhu S Ma L Xiang B . Systematic review comparing the safety and efficacy of conventional and drug-eluting bead transarterial chemoembolization for inoperable hepatocellular carcinoma. Hepatol Res. (2015) 45:190–200. doi: 10.1111/hepr.12450, PMID: 25388603

[20] Huang K Zhou Q Wang R Cheng D Ma Y . Doxorubicin-eluting beads versus conventional transarterial chemoembolization for the treatment of hepatocellular carcinoma. J Gastroenterol Hepatol. (2014) 29:920–5. doi: 10.1111/jgh.12439, PMID: 24224722

[21] Chinese Society of Hepatology, Chinese Medical Association Chinese Society of Infectious Diseases, Chinese Medical Association . Guidelines for the prevention and treatment of chronic hepatitis B (2022 version). Chin J Hepatol. (2022) 30:1309–31. doi: 10.3760/cma.j.cn501113-20221224-00607 PMC12677433 36891718

[22] Deng H Kan A Lyu N He M Huang X Qiao S . Tumor-derived lactate inhibit the efficacy of lenvatinib through regulating PD-L1 expression on neutrophil in hepatocellular carcinoma. J Immunother Cancer. (2021) 9:e002305. doi: 10.1136/jitc-2020-002305, PMID: 34168004 PMC8231064

[23] Wu J-Y Yin Z-Y Bai Y-N Chen Y-F Zhou S-Q Wang S-J . Lenvatinib combined with anti-PD-1 antibodies plus transcatheter arterial chemoembolization for unresectable hepatocellular carcinoma: a multicenter retrospective study. J Hepatocell Carcinoma. (2021) 8:1233–40. doi: 10.2147/JHC.S332420, PMID: 34676181 PMC8502053

[24] Zhang L Geng Z Hao B Geng Q . Tislelizumab: A modified anti-tumor programmed death receptor 1 antibody. Cancer Control. (2022) 29:10732748221111296. doi: 10.1177/10732748221111296, PMID: 35926155 PMC9358212

[25] Qin S Kudo M Meyer T Bai Y Guo Y Meng Z . Tislelizumab vs sorafenib as first-line treatment for unresectable hepatocellular carcinoma: A phase 3 randomized clinical trial. JAMA Oncol. (2023) 9:1651–9. doi: 10.1001/jamaoncol.2023.4003, PMID: 37796513 PMC10557031

[26] Zheng JR Wang ZL Feng B . Hepatitis B HBsAg clearance and immune response. Front Immunol. (2022) 13:1075916. doi: 10.3389/fimmu.2022.1075916, PMID: 36466821 PMC9714500

[27] Xiao G Ren H . Patients with HCC as an ideal study population to assess immune checkpoint inhibitors in hepatitis B. J Hepatol. (2025) 82:e137–8. doi: 10.1016/j.jhep.2024.10.010, PMID: 39423866

[28] Hoogeveen RC Boonstra A . Checkpoint inhibitors and therapeutic vaccines for the treatment of chronic HBV infection. J Hepatol. (2020) 73:1431–43. doi: 10.3389/fimmu.2020.00401, PMID: 32194573 PMC7064714

[29] Su M Ye T Wu W Shu Z Xia Q . Possibility of PD-1/PD-L1 inhibitors for the treatment of patients with chronic hepatitis B infection. Dig Dis. (2024) 42:53–60. doi: 10.1159/000534535, PMID: 37820605 PMC10836741

[30] Li B Yan C Zhu J Chen X Fu Q Zhang H . Anti–PD-1/PD-L1 blockade immunotherapy employed in treating hepatitis B virus infection–related advanced hepatocellular carcinoma: A literature review. Front Immunol. (2020) 11:1037. doi: 10.3389/fimmu.2020.01037, PMID: 32547550 PMC7270402

[31] Shao YY Chen CT Chuang CH Su TH Ho MC Tseng TC . Prompt initiation of durvalumab and tremelimumab for unresectable hepatocellular carcinoma in patients with chronic active hepatitis B: a phase 2 clinical trial. Br J Cancer. (2025) 132:822–7. doi: 10.1038/s41416-025-02978-7, PMID: 40128285 PMC12041533

[32] Zhou J Chen G Wang J Zhou B Sun X Wang J . Anti-PD-1 therapy achieves favorable outcomes in HBV-positive non-liver cancer. Oncogenesis. (2023) 12:22. doi: 10.1038/s41389-023-00468-0, PMID: 37080999 PMC10119302

[33] Mon HC Lee PC Hung YP Hung YW Wu CJ Lee CJ . Functional cure of hepatitis B in patients with cancer undergoing immune checkpoint inhibitor therapy. J Hepatol. (2025) 82:51–61. doi: 10.1016/j.jhep.2024.07.018, PMID: 39084471

[34] Ding ZN Meng GX Xue JS Yan LJ Liu H Yan YC . Hepatitis B virus reactivation in patients undergoing immune checkpoint inhibition: systematic review with meta-analysis. J Cancer Res Clin Oncol. (2023) 149:1993–2008. doi: 10.1007/s00432-022-04133-8, PMID: 35767193 PMC11797397

[35] L’Orphelin JM Da Silva A Cabon J Alexandre J Dolladille C . Immune checkpoint inhibitor rechallenge after immune-related adverse events: a retrospective study from VigiBase update in 2024 looking for emergent safety signals. BMJ Open. (2024) 14:e091708. doi: 10.1136/bmjopen-2024-091708, PMID: 39627133 PMC11624719

[36] Le Guennec L Mouri S Thabut D Weiss N . Blood-brain barrier dysfunction in hepatic encephalopathy: pathophysiology, diagnostic assessment and therapeutic perspectives. Metab Brain Dis. (2025) 40:223. doi: 10.1007/s11011-025-01645-3, PMID: 40512384

[37] Liang X Liu H Chen H Peng X Li Z Teng M . Rhein-based Pickering emulsion for hepatocellular carcinoma: Shaping the metabolic signaling and immunoactivation in transarterial chemoembolization. Aggregate. (2024) 5:e552. doi: 10.1002/agt2.552

[38] Ji JH Ha SY Lee D Sankar K Koltsova EK Abou-Alfa GK . Predictive biomarkers for immune-checkpoint inhibitor treatment response in patients with hepatocellular carcinoma. Int J Mol Sci. (2023) 24:1–27. doi: 10.3390/ijms24087640, PMID: 37108802 PMC10144688

